# Prognostic value of pretreatment serum beta-2 microglobulin level in advanced classical Hodgkin lymphoma treated in the modern era

**DOI:** 10.18632/oncotarget.12663

**Published:** 2016-10-14

**Authors:** Qin Wang, Yan Qin, Shengyu Zhou, Xiaohui He, Jianliang Yang, Suyi Kang, Peng Liu, Sheng Yang, Changgong Zhang, Lin Gui, Yan Sun, Yuankai Shi

**Affiliations:** ^1^ Department of Medical Oncology, Beijing Key Laboratory of Clinical Study on Anticancer Molecular Targeted Drugs, National Cancer Center/Cancer Hospital, Chinese Academy of Medical Sciences and Peking Union Medical College, Beijing 100021, China

**Keywords:** classical Hodgkin lymphoma, prognosis, international prognostic score, serum beta-2 macroglobulin

## Abstract

The prognostic value of pretreatment serum beta-2 microglobulin (B2MG) level in advanced Hodgkin lymphoma (HL) patients treated in the modern era has not been well established. We conducted a retrospective study involving 202 advanced classical HL (cHL) patients treated from 1998.5 to 2015.7 to evaluate the impact of serum B2MG level on prognosis. Multivariate analysis showed that serum B2MG level ≥ 2.5 mg/L was an independent predictor for freedom from progression (FFP) (*P* = 0.001), lymphoma-specific survival (*P* = 0.030) and overall survival (*P* = 0.034). The 5-year FFP of patients with serum B2MG level ≥ 2.5 mg/L was 66.8%, compared with 89.7% in patients with B2MG level < 2.5 mg/L (*P* < 0.001). The traditionally used International Prognostic Score (IPS) remained prognostic for FFP (*P* = 0.013) but the predictive range narrowed, with 5-year FFP ranging from 90.9% to 62.3%. The 5-year FFP of the 44 patients with both IPS ≥ 3 and serum B2MG ≥ 2.5 mg/L was 50.7%, which was significantly worse than that of the 87 patients with only one of the two factors (81.9%, *P* < 0.001) or the 71 patients with both B2MG < 2.5 mg/L and IPS < 3 (91.1%, *P* < 0.001). The difference of FFP between the latter two groups was smaller but also significant (*P* = 0.038). In summary, our data suggest pretreatment serum B2MG level ≥ 2.5 mg/L was an independent unfavorable prognostic factor in advanced cHL patients treated in the modern era. It improves IPS in predicting the outcomes as the combination of IPS and B2MG indentified a wider prognostic range than IPS alone with a sizable number of patients in different risk groups.

## INTRODUCTION

Hodgkin lymphoma (HL) is a curable disease, but about 20–30% of advanced patients will suffer relapse [1] and some lower risk patients may be overtreated, which result in an increased toxicity during the long-term survival [2, 3]. It is highly important to identify patients with different prognosis accurately so as to guide decisions of individualized risk-adapted therapy.

The International Prognostic Score (IPS) [4] was a seven-factor prognostic scoring system developed in 1998 based on data of patients treated before 1992, which was widely used to guide treatment decisions in clinical practice since then. However, the IPS had decreased utility in patients treated in the modern era with an obviously narrowed predictive range [5, 6]. In recent years, there has been an increasing tendency of investigating new prognostic factors for HL [7–9].

Serum beta-2 microglobulin (B2MG), a small molecule protein, has been found elevated and associated with prognosis in many lymphoid malignancies [10–13], such as follicular lymphoma, NK/T-cell lymphoma, diffuse large B cell lymphoma (DLBCL) and mantle cell lymphoma. There were a few studies exploring the prognostic value of pretreatment serum B2MG level in HL [14–18], of which either the first-line treatments were not uniform or the sample size was relatively small. Additional data are still necessary to confirm its role in HL. Furthermore, nobody ever analyzed the prognostic effect of B2MG level in advanced HL separately or assessed the combined prognostic value of IPS and B2MG level for patients in the modern era.

Β2MG has been routinely detected in clinical practice in our hospital since the end of the 20th century. We collected the data of advanced classical HL (cHL) patients treated in recent decades to assess the impact of pretreatment serum Β2MG level on prognosis retrospectively.

## RESULTS

### Patient characteristics

The baseline characteristics of patients were summarized in Table 1. The median age at diagnose was 37 years old (range, 15–65 years) and 44 cases (21.8%) were more than 45 years old. More than half of the patients (54.0%) were male and 87 cases (43.1%) had stage IV disease. The two most common pathological types were Nodular sclerosis cHL (48.5%) and mixed cellularity cHL (34.7%). Nearly half of the cases (48.5%) received chemoradiotherapy as first-line treatment. The three hematological index of IPS (Hemoglobin < 10.5g/dL, white blood cell ≥ 15 × 10^9^/L, Lymphocyte < 0.6 × 10^9^/L or < 8% of white blood cell) were present in about 20% of the patients and less than half of the patients has a serum albumin < 4 g/dL (44.6%). Patients with IPS ≥ 5 were considered as a group for only 6 patients had a score ≥ 6.

**Table 1:** Baseline characteristics of 202 patients and correlation of serum B2MG level (mg/L) with other parameters

Characteristics	No. *n* = 202 (%)	B2MG ≥ 2.5 *n* = 99 (49.0%)	B2MG < 2.5 *n* = 103 (51.0%)	*P* value
Age median(range)	37 (15–65)	39 (17–65)	34 (15–65)	0.005
Stage				0.010
II	21 (10.4)	7 (7.1)	14 (13.6)	
III	94 (46.5)	39 (39.4)	55 (53.4)	
IV	87 (43.1)	53 (53.5)	34 (33.0)	
B symptoms	97 (48.0)	56 (56.6)	41 (39.8)	0.017
Histopathology				0.643
Nodular sclerosis	98 (48.5)	48 (48.5)	50 (48.5)	
Mixed cellularity	70 (34.7)	33 (33.3)	37 (35.9)	
Lymphocyte-rich	17 (8.4)	7 (7.1)	10 (9.7)	
Lymphocyte-depleted	7 (3.5)	4 (4.0)	3 (2.9)	
Unclassified	10 (5.0)	7 (7.1)	3 (3.9)	
Bulky disease	46 (22.8)	25 (25.3)	21 (20.4)	0.410
Elevated LDH	77 (38.1%)	45 (45.5)	32 (31.1)	0.035
Number of extranodal sites				
0–1	162 (80.2)	76 (76.8)	86 (83.5)	0.230
≥ 2	40 (19.8)	23 (23.3)	17 (16.5)	
Primary treatment				0.568
Chemotherapy	104 (51.5)	54 (54.5)	50 (48.5)	0.394
Chemoradiotherapy	98 (48.5)	45 (45.5)	53 (51.5)	
IPS risk factors				
Male	109 (54.0)	55 (55.6)	54 (52.4)	0.656
Age ≥ 45 yr	44 (21.8)	28 (28.3)	16 (15.5)	0.028
StageIV	87 (43.1)	53 (53.5)	34 (33.0)	0.003
Hemoglobin < 10.5 g/dL	40 (19.8)	27 (27.3)	13 (12.6)	0.009
Serum albumin < 4 g/dL	90 (44.6)	46 (46.5)	44 (42.7)	0.592
WBC ≥ 15 × 10^9^/L	41 (20.3)	28 (28.3)	13 (12.6)	0.006
Lymphocytopenia	37 (18.3)	25 (25.3)	12 (11.7)	0.012
IPS score				< 0.001
0	18 (8.9)	5 (5.1)	13 (12.6)	
1	46 (22.8)	12 (12.1)	34 (33.0)	
2	62 (30.7)	38 (38.4)	24 (23.3)	
3	44 (21.8)	17 (17.2)	27 (26.2)	
4	21 (10.4)	19 (19.2)	2 (1.9)	
≥ 5	11 (5.4)	8 (8.1)	3 (2.9)	

Note: B2MG = beta-2 microglobulin, LDH = lactate dehydrogenase, IPS = International Prognostic Score, WBC = white blood cell, Lymphocytopenia = Lymphocyte < 0.6 × 10^9^/L or < 8% of WBC.

### Outcomes

At a median follow-up of 85 months (range 12–218 months), forty-five patients progressed or relapsed and 29 patients died: 25 died of HL,1 died from pneumonia, 1died of acute myeloid leukemia transformed from myelodysplastic syndrome, and 2 were due to second primary malignancy(1 lung cancer and 1 breast cancer). One patient was lost to follow-up. The 5-year FFP, 5-year OS, and 5-year LSS was 78.6%, 88.3% and 90.2% respectively.

### Cutoff value of serum B2MG level

**Figure 1:**
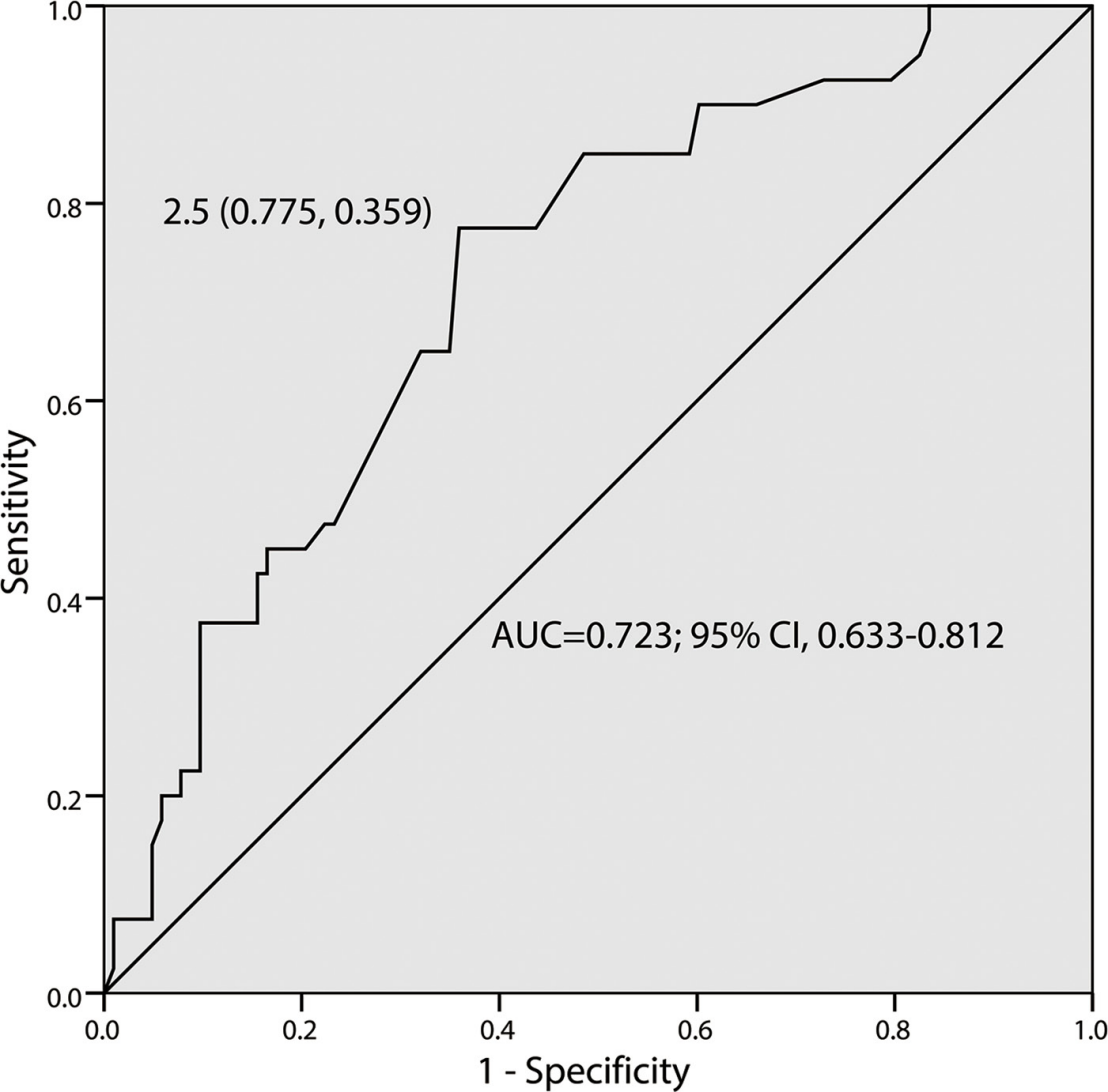
Receiver operating characteristic (ROC) curve and area under the curve (AUC) for pretreatment serum beta 2-microglobulin (B2MG) level

ROC curve was generated to determine the best cutoff value for the pretreatment serum B2MG level. The area under the curve (AUC) was recorded as 0.723 (95% CI, 0.633–0.812) (Figure 1). The value of 2.5 mg/L corresponded to the maximum joint sensitivity and specificity on the ROC curve (77.5% sensitivity and 64.1% specificity), which was defined as the cutoff value. Ninety-nine (49.0%) patients had a baseline serum B2MG level ≥ 2.5 mg/L and the remaining patients had a serum B2MG level < 2.5 mg/L.

### Association between serum B2MG level and other characteristics

The correlation of the pretreatment serum B2MG level and other baseline characteristics was also summarized in Table 1. A serum B2MG level ≥ 2.5 mg/L was significantly correlated with an older age (*P* = 0.005), a higher Ann Arbor stage (*P* = 0.010), the presence of B symptoms (*P* = 0.017), elevated lactate dehydrogenase (LDH) (*P* = 0.035) and the constituent ratios of IPS scores were different in the two groups (*P* < 0.001). As for the seven IPS factors, a serum B2MG level ≥ 2.5 mg/L was significantly associated with age ≥ 45 years (*P* = 0.028), stage IV (*P* = 0.003), hemoglobin < 10.5g/dL (*P* = 0.009), WBC ≥ 15 × 10^9^/L (*P* = 0.006) and lymphocyte < 0.6 × 10^9^/L or < 8% of WBC (*P* = 0.012). No significant correlation was observed between serum B2MG level and histological type (*P* = 0.643), bulky disease (*P* = 0.410), number of extranodal sites (*P* = 0.230), sex (*P* = 0.656) and serum albumin < 4 g/dL (*P* = 0.592). Besides, there is no significant distinction in primary treatment (chemotherapy alone or chemoradiotherapy) between the two groups (*P* = 0.394).

### Prognostic analysis

The prognostic significance of baseline parameters for FFP, LSS and OS evaluated by the Cox proportional hazard regression analysis was shown in Table 2. In univariate analysis, factors negatively correlated with FFP included elevated LDH (*P* = 0.018), IPS ≥ 3 (*P* = 0.001) and serum B2MG level ≥ 2.5 mg/L (*P* < 0.001). IPS ≥ 3 and serum B2MG level ≥ 2.5 mg/L were also prognostic for LSS (*P* = 0.025, 0.020) and OS (*P* = 0.048, 0.026). In multivariate analysis, IPS ≥ 3 and B2MG level ≥ 2.5 mg/L have significant independent negative prognostic value for FFP (HR = 2.475, 95%CI, 1.230–4.978, *P* = 0.011; HR = 3.223, 95%CI, 1.651–6.292, *P* = 0.001) and LSS (HR=2.316, 95%CI, 1.037–5.176, *P* = 0.041; HR=2.633, 95%CI, 1.096–6.326, *P* = 0.030). Only B2MG level ≥ 2.5 mg/L was independent prognostic factor for OS (HR = 2.343, 95%CI, 1.064–5.159, *P* = 0.034).

**Table 2:** Univariate and mutivariate analysis for freedom-from progression (FFP), lymphoma-specific survival (LSS) and overall survival (OS)

Variables	FFP			LSS			OS		
	HR	95% CI	*P*	HR	95% CI	*P*	HR	95% CI	*P*
**Univariate Analysis**									
Bulky disease	1.433	0.752–2.731	0.274	1.638	0.707–3.798	0.250	1.364	0.602–3.092	0.457
B symptoms	1.545	0.856–2.787	0.148	1.896	0.849–4.234	0.119	1.385	0.666–2.879	0.383
Number of extranodal sites	1.576	0.814–2.052	0.177	1.680	0.701–4.026	0.245	1.409	0.600–3.312	0.431
Elevated LDH	2.028	1.129–3.643	**0.018**	1.514	0.691–3.320	0.300	1.363	0.655–2.837	0.408
IPS ≥ 3	2.798	1.541–5.082	**0.001**	2.494	1.120–5.554	**0.025**	2.099	1.007–4.373	**0.048**
B2MG ≥ 2.5 mg/L	3.483	1.797–6.750	**< 0.001**	2.821	1.177–6.671	**0.020**	2.455	1.116–5.399	**0.026**
Chemoradiotherapy	1.117	0.621–2.009	0.712	1.327	0.593–2.967	0.491	1.059	0.507–2.212	0.879
**Mutivariate Analysis**									
LDH	1.082	0.542–2.157	0.824	—	—	—	—	—	—
IPS ≥ 3	2.475	1.230–4.978	**0.011**	2.316	1.037–5.176	**0.041**	1.988	0.951–4.156	0.068
B2MG ≥ 2.5 mg/L	3.223	1.651–6.292	**0.001**	2.633	1.096–6.326	**0.030**	2.343	1.064–5.159	**0.034**

Note: HR = Hazard ratio, CI = confidence interval, LDH = lactate dehydrogenase, IPS: International Prognostic Score, B2MG: beta-2 microglobulin.

**Table 3:** Estimated 5-year freedom from progression (FFP) according to the International Prognostic Score (IPS)

IPS	5-FFP	
	Our study	Original IPS study
0	88.5%	84%
1	90.9%	77%
2	81.5%	67%
3	68.7%	60%
4	63.5%	51%
≥ 5	62.3%	42%

### Combined prognostic value of IPS and serum B2MG level

IPS remain prognostic for FFP (*P* = 0.013) of the 202 patients in our study, but the magnitude narrowed obviously compared with the original IPS study, with 5-year FFP ranging from 90.9% to 62.3% (Table 3). Even the 5-year FFP of patients with IPS = 0(88.5%) was lower than that of patients with IPS = 1(90.9%).

**Figure 2:**
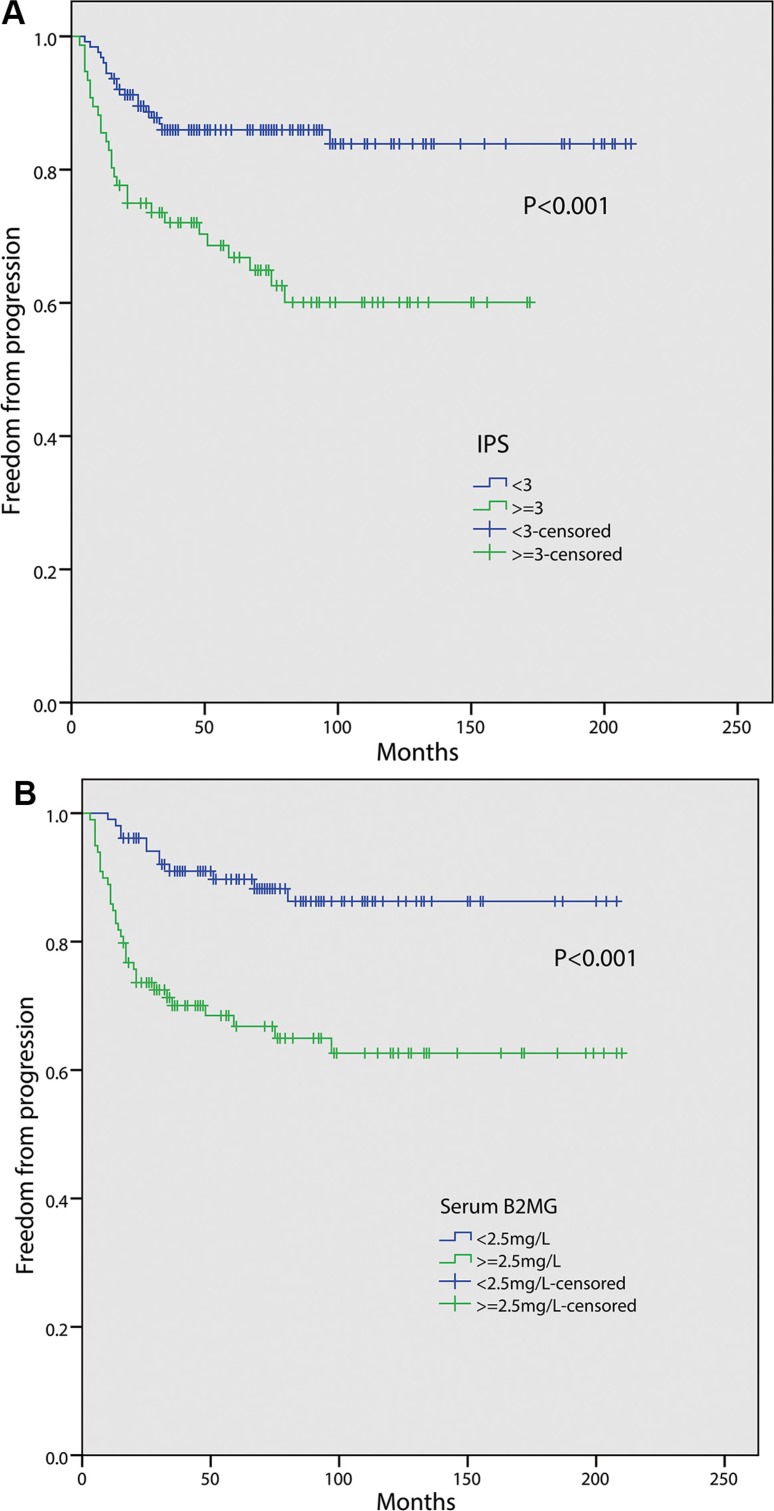
Freedom from progression (FFP) according to: International Prognostic Score (IPS) (< 3 vs. ≥ 3) (A); pretreatment serum beta 2-microglobulin (B2MG) level (< 2.5 mg/L vs. ≥ 2.5 mg/L) (B).

**Figure 3:**
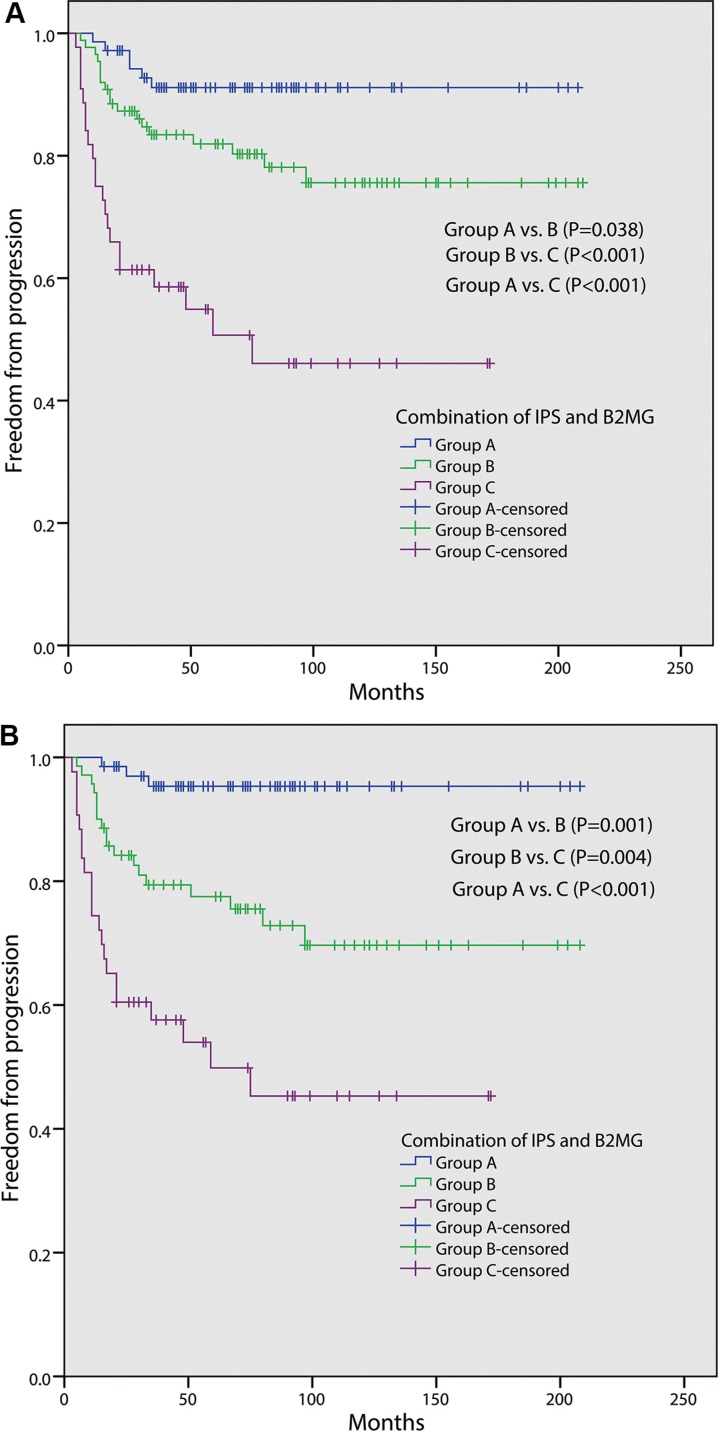
Freedom from progression (FFP) according to the combination of International Prognostic Score (IPS) and serum beta-2 microglobulin (B2MG) level in 202 patients (A); in 181 patients with stage III/IV (B). Group A: both B2MG < 2.5 mg/L and IPS < 3; Group B: either B2MG ≥ 2.5 mg/L or IPS ≥ 3; Group C: both B2MG ≥ 2.5mg/L and IPS ≥ 3

Patients with IPS ≥ 3 have a poor FFP (5-year FFP, 66.8%) compared with those with IPS < 3 (86.0%, *P* < 0.001, Figure 2A). The FFP of patients with serum B2MG level ≥ 2.5 mg/L was significantly worse than those with B2MG level < 2.5 mg/L (5-year FFP rate, 66.8% vs. 89.7%, *P* < 0.001, Figure 2B). To assess the combined prognostic value of serum B2MG level and IPS, we separated the 202 patients into three groups: 71(35.1%) patients with both B2MG < 2.5 mg/L and IPS < 3 (group A), 87(43.1%) patients with either B2MG ≥ 2.5 mg/L or IPS ≥ 3 (group B), 44(21.8%) patients with both B2MG ≥ 2.5 mg/L and IPS ≥ 3 (group C). As illustrated in Figure 3A, group C has a significantly inferior FFP (5-year FFP, 50.7%) compared with group A (5-year FFP, 91.1%, *P* < 0.001) or group B (5-year FFP, 81.9%, *P* < 0.001). The difference of FFP between the latter two groups was smaller but also significant (*P* = 0.038). For the 181 patients with stage III/IV, 68(37.6%), 70(38.7%) and 43(23.8%) patients belong to group A, B and C respectively and the 5-year FFP was also different between every two groups (group A vs. group B, 95.3% vs. 77.5%, *P* = 0.001; group B vs. group C, 77.5% vs. 49.8%, *P* = 0.004; group A vs. group C, 95.3% vs. 49.8%, *P* < 0.001; Figure 3B).

## DISCUSSION

In our study, elevated pretreatment serum B2MG level showed independent unfavorable prognostic value in advanced cHL patients treated in the modern era. It improves IPS in predicting the outcomes as the combination of IPS and B2MG indentified a wider prognostic range than IPS alone with a sizable number of patients in different risk groups.

There exist some studies [14–18] before which reported the adverse prognostic value of serum B2MG level in HL, but these studies included patients of all stages or only early stage, who were treated with different first-line chemotherapy regimens. Only one study [18] in the 21st century enrolled patients receiving a uniform regimen (ABVD) combined with or without radiotherapy, but it had a sample size of 67 and the numbers of chemotherapy cycle of were different in these patients. Most of the previous studies [14–17] used the upper normal limits of serum B2MG as cutoff value. Our study used cutoff value based on ROC curves, which corresponded to the maximum joint sensitivity and specificity. Furthermore, cHL account for a large proportion of HL, which has a very different natural history or therapy compared with nodular lymphocyte predominance HL [19]. Nobody ever evaluated the prognostic significance of serum B2MG level in cHL separately. The 202 advanced cHL patients in our study received 6 cycles of ABVD+/−radiotherapy as first-line treatment. The Chi-square test suggested there was no significant distinction in primary treatment (chemotherapy alone or chemoradiotherapy) between the two B2MG level groups (Table 1), which reduced the bias caused by the heterogeneity of treatment.

Besides, different from these studies mentioned above, we included patients with a narrower age range (15–65 years) instead of any age, which coincides with the age range of the original IPS study [4]. Some investigators suggested the prognosis of elderly Hodgkin lymphoma should be analyzed separately since the survival of this special population has been found disproportionately inferior compared with younger patients mainly because of their inadequate treatment resulting from poor tolerance [20–22].

In our study, elevated serum B2MG level was significantly correlated with higher Ann Arbor stage, the presence of B symptoms, elevated LDH, older age, hemoglobin < 10.5 g/dL, WBC ≥ 15 × 10^9^/L and lymphocytopenia (Table 1), most of which are the conventional poor prognostic factors of HL [4]. These findings are similar to that reported previously [14–18], even if there is not too much comparability between those studies and ours.

IPS was developed based on data of patients treated before 1992, which predict 5-year FFP from 84% to 42% [4]. Some investigators found the utility of IPS was limited in patients treated in the modern era [5, 6], which was mainly due to the improved outcomes. Similarly, in our study, the IPS remained prognostic for FFP but the predictive range narrowed obviously (90.9%–62.3%). The combination of B2MG level and IPS identified a wider prognostic range (5-year FFP, 91.1% to 50.7%), especially in patients with stage III/IV (5-year FFP, 95.3% to 49.8%). In addition, the FFP survival curves of the three groups divided according to the two factors separate from each other very well.

B2MG is synthesized in almost all nucleated cells and constitutes the light chain subunit of the human leukocyte antigen-I (HLA-I), which is distributed on cellular membrane [23]. It is released into the body fluids such as serum during cell turnover and is excreted mainly via the kidneys [24]. The mechanisms underlying the poor prognostic significance of an elevated B2MG level for lymphomas remains to be elucidated. Investigators conducting studies in DLBCL [12] speculated that higher serum B2MG levels correlates with the absence of HLA-I expression, which could lead to a defective recognition of tumor-specific antigens by T-cells [25]. Whether this theory apply to HL needs investigation.

In conclusion, our study suggested that pretreatment serum B2MG, a routine-detected index in clinical practice, was of great prognostic value in advanced cHL and the combination of IPS and B2MG confers stronger prognostic value than IPS alone. A multi-center study with larger group of patients and longer follow-up times is needed to validate our findings.

## MATERIALS AND METHODS

### Patients

Among all the advanced HL patients consecutively admitted to Cancer Hospital Chinese Academy of Medical Sciences (CHCAMS)from 1998.5 to 2015.7, 202 individuals met the inclusion criteria of this study: histologic and imaging confirmation of advanced classical HL (defined as stage III/IV or stage II with bulky disease) with an age range of 15–65 years; previously untreated; no history of malignancy; Six cycles of ABVD with or without radiotherapy (applied to bulky tumors or to sites of residual disease) as first-line therapy; clinical data were complete and available from the hospital medical records. The exclusion criteria are as follows: cardiopulmonary or hepatic or renal dysfunction; pregnancy; immunosuppression; immunodeficiency; immunosuppressive medications users; history of transplantation. Patients’ baseline clinical and laboratory parameters were collected. Frequency of follow-up back to hospital after treatment is as follows: every 3 months for the first 2 years, every 6 months for the next 3 years and then annually or whenever clinically indicated after 5 years. We got all the information we need from the case management system in the medical record room of CHCAMS. This study was in compliance with the Declaration of Helsinki and was approved by the Medical Ethics Committee of CHCAMS. Patient records was anonymized and de-identified prior to analysis.

### Statistical analysis

FFP [26] was defined as the interval from the date of diagnosis to the first recurrence (progression or relapse) of disease with deaths not caused by disease progression/relapse during remission or loss to follow-up censored. Lymphoma-specific survival (LSS) [26] was defined as the interval from the date of diagnosis to the death as a result of lymphoma with deaths unrelated to lymphoma or loss to follow-up censored. Overall survival (OS) [26] was defined as the interval from the date of diagnosis to the death from any cause with loss to follow-up censored. The treatment regimens in progressed or recurrent cases are various and salvage therapy could rescue many relapsed patients, so the prognostic value of serum B2MG level for FFP was regarded as the primary end point. Receiver operating characteristic (ROC) curve analysis was used to determine the optimal cutoff values of B2MG. The binary clinical outcome was determined at 5 years after diagnosis. FFP, LSS and OS were calculated with Kaplan-Meier methodology and compared using log-rank testing. The Chi-square test or Fisher's exact test was performed to compare the distribution of patient characteristics between high and low level B2MG groups for categorical variables and *t* test for continuous variables if the data followed normal distribution. The Cox proportional hazard model was used for the univariate and multivariate analyses to evaluate the prognostic effect of parameters for survival, which was expressed as hazard ratio (HR) with a 95% confidence interval (CI). All *p value*s reported were two-sided with *p* < 0.05 considered statistically significant. The statistical analyses were performed using SPSS version 22.0 statistical software (SPSS Inc., Chicago, IL, USA).
